# Trends and Mortality Predictors of Delirium Among Hospitalized Older Adults: A National 5-Year Retrospective Study in Thailand

**DOI:** 10.3390/geriatrics10040088

**Published:** 2025-07-01

**Authors:** Manchumad Manjavong, Panita Limpawattana, Jarin Chindaprasirt, Poonchana Wareechai

**Affiliations:** 1 Department of Internal Medicine, Faculty of Medicine, Khon Kaen University, Khon Kaen 40002, Thailand; manchu@kku.ac.th (M.M.); jarich@kku.ac.th (J.C.); 2 National Health Security Office, Bangkok 10210, Thailand; poonchana.w@nhso.go.th

**Keywords:** clinical epidemiology, confusion, delirium, elderly

## Abstract

Background: Delirium frequently manifests in hospitalized geriatric patients and is associated with negative health outcomes. Available large-scale data regarding its prevalence rate and impact on older Thai patients are limited. This study aimed to analyze trends in the prevalence rate, its consequences, and the factors contributing to death at discharge among this population. Methods: A retrospective study of inpatients over the age of 60 who received a diagnosis of delirium was conducted, utilizing inpatient medical expense documentation for the fiscal years 2019–2023. The identification of delirium was conducted by the National Health Security Office using the International Statistical Classification of Diseases and Related Health Problems, 10th Revision, Thai Modification (ICD-10-TM) code F05. Results: The 5-year prevalence rate and mortality rate of delirium were 215.1 and 18.7/100,000 population, respectively, and tended to rise over the studied periods. The average hospitalization was 10 days, and the average healthcare expenditure was about 1470 USD/visit. Respiratory disease emerged as the most common primary diagnosis in delirious patients (23.5%). Factors associated with mortality were individuals aged >80 years when juxtaposed with the cohort aged 61–70 years (adjusted odds ratio [AOD] 1.07), being female (AOR 1.13), and a primary diagnosis of respiratory disease (AOR 2.72), cardiovascular disease (AOR 1.68), musculoskeletal disease (AOR 0.61), systemic infection/septicemia (AOR 2.08); or malignancy (AOR 2.97). Conclusions: There was an upward trend in rates of both prevalence and mortality associated with delirium among hospitalized geriatric patients. Advancing age, gender, and particular primary diagnoses were associated with mortality at hospital discharge.

## 1. Introduction

Delirium is an acute neuropsychiatric disorder characterized by abrupt alterations in attention, consciousness, and cognitive abilities. This condition is frequently observed in hospitalized elderly patients, and its prevalence varies across diverse studies [1,2]. A systematic review and meta-analysis encompassing 48 studies from medical, surgical, or specialty intensive care units revealed an overall pooled prevalence of 31%, with even higher rates observed in patients requiring mechanical ventilation, fluctuating between 60% and 80% [3]. The etiology of this condition is multifactorial, categorized into two principal groups: predisposing factors and precipitating factors. Predisposing factors encompass advanced age, male gender, preexisting neurological disorders such as dementia and Parkinson’s disease, dependency in functional status, co-existing medical conditions, and visual and auditory impairments. Conversely, precipitating factors include pharmacological interventions, primary neurological disorders, concurrent illnesses, surgical procedures, changes in bed assignment, extended periods of sleep deprivation, immobility, uncontrolled pain, and the use of physical restraints [3,4].

Delirium constitutes a significant medical concern, as it correlates with numerous adverse health outcomes, including elevated healthcare costs, extended duration of hospitalization, considerable cognitive impairment and dependency both in the short and long term, and increased mortality [5,6]. The diagnosis of delirium predominantly relies on clinical assessment and established diagnostic criteria. Consequently, the prompt identification of predisposing factors and the avoidance of precipitating factors by healthcare professionals, when possible, are imperative, given their potential for prevention as a medical condition [1,2].

A large-scale data study investigating prevalence rates and their implications among older Thai patients was conducted in 2010. This study indicated that delirium was identified in 11,410 of all prevalence, contributing to prevalence and mortality rates of 155.4 and 6.4 per 100,000 older individuals, respectively. These figures escalated with advancing age [7]. Since that time, multiple studies and hospital policies have emerged that emphasize the significance of practical diagnostic instruments, such as the Thai version of the Confusion Assessment Method (CAM) [8,9] and the 4AT test [10], alongside effective management strategies for delirium [11,12]. There is a paucity of studies addressing the occurrence of delirium and its clinically relevant outcomes. Therefore, this research aimed to examine the trends in prevalence rates, mortality rates, length of hospital stay, and healthcare expenditures associated with delirium over the preceding 5 years (2019–2023). Additionally, we evaluated the predictors linked to mortality at hospital discharge in this population. While delirium is a grave condition associated with elevated mortality rates, it is vital to recognize that not all patients diagnosed with delirium will experience identical outcomes. The identification of high-risk patients and the implementation of timely interventions may potentially reduce these risks.

## 2. Methods

### 2.1. Study Design and Populations

This retrospective analysis utilized data extracted from inpatient medical expense documentation for the fiscal period spanning from 2019 to 2023 (specifically from 1 October 2018 to 30 September 2023). The dataset was retrieved from the National Health Security Office by employing the International Statistical Classification of Diseases and Related Health Problems, 10th Revision, Thai Modification (ICD-10-TM), which encompassed all inpatients over the age of 60 years diagnosed with delirium (ICD-10-F05), classified as either a primary or secondary diagnosis. Therefore, this study included all geriatric patients who developed delirium at hospital admission or during hospitalization.

### 2.2. Data Collections

Data collection included the patient’s age, gender, month and year of admission and discharge, primary diagnosis (in case which delirium was not primary diagnosis), hospital length of stay (LOS), healthcare cost, and discharge status.

### 2.3. Statistical Analyses

The demographic characteristics of the patients were delineated utilizing descriptive statistics. Prevalence and mortality rates within the hospital setting for patients with delirium per 100,000 individuals were calculated across the total population. Adjustment for age was executed through the standardization to the demographic data from 2019–2023, utilizing a direct methodology that incorporated the weighting of age-specific rates identified within the population according to the proportional representation of each age group (61–70, 71–80, and 80 years or over) in a standard reference population [13]. Factors correlated with mortality at discharge were analyzed through univariate methods and articulated as crude odds ratios (OR) accompanied by a 95% confidence interval (CI). Variables exhibiting *p*-values of less than 0.2 were subsequently included in the multiple logistic regression analysis after checking for multicollinearity. The rationale for using a *p*-value threshold of less than 0.2 from univariate analysis was to avoid the premature exclusion of important variables [14]. Variables with *p*-values less than 0.05 were deemed statistically significant and reported as adjusted odds ratios (OR) with a 95% confidence interval (CI).

## 3. Results

### 3.1. Prevalence Rates of Delirium

**Figure 1:**
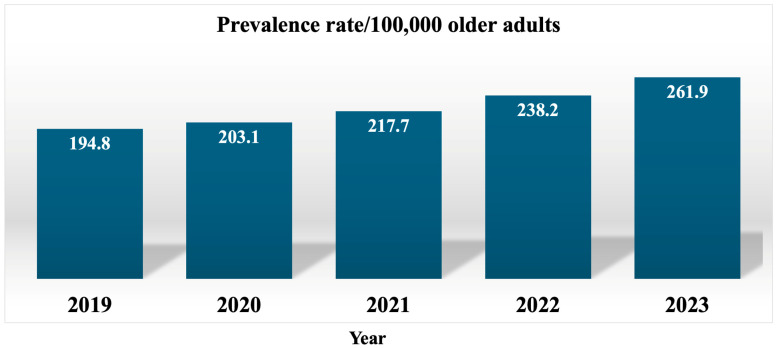
Prevalence rates of older patients with delirium per 100,000 older adults from 2019–2023.

Delirium was diagnosed in 160,375 visits among geriatric patients during the fiscal years spanning from 2019 to 2023, contributing to an overall 5-year prevalence rate of 215.1 per 100,000 older individuals, with an average duration of hospitalization of 10 days and an average healthcare expenditure of 50,060.9 THB per visit (approximately 1470 USD per visit). The average age of the patients was 77.3 years, with a standard deviation of 8.6 years (median age of 78 years, interquartile ranges of 71–84 years), and most patients were female (51.5%). The predominant four primary diagnoses of these patients included respiratory tract disease (23.5%), followed by neurological disease (13.8%), genitourinary disease (12.3%), and metabolic disease (12.3%), in that order. Additional primary diagnoses comprised diseases of the gastrointestinal tract (10.0%), the cardiovascular system (9.8%), musculoskeletal disease (8.6%), malignancy (5.6%), and systemic infection or septicemia (4.1%). The annual prevalence rate per 100,000 older individuals is illustrated in Figure 1. The prevalence rate, duration of stay, and healthcare costs stratified by age group are presented in Table 1. It was observed that greater age was associated with a greater prevalence rate of delirium, with a reduced length of hospital stay and diminished healthcare expenditures during the period of hospitalization.

### 3.2. Mortality Rate at Discharge, Hospital Stay, and Healthcare Costs of Inpatients Diagnosed with Delirium

**Figure 2:**
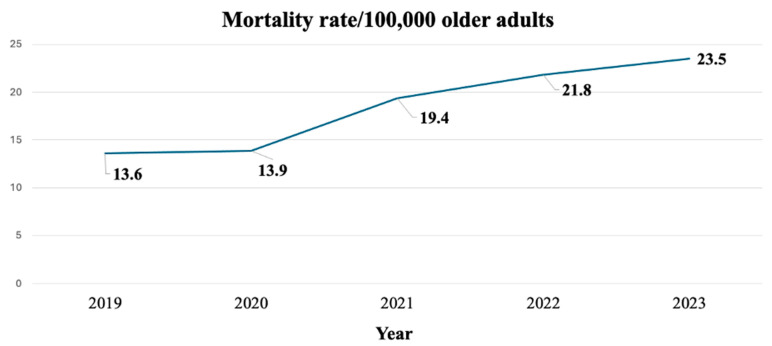
Mortality rates of older patients with delirium per 100,000 older adults from 2019–2023.

The overall 5-year mortality rate per 100,000 older persons from 2019–2023 was 18.7, with the average LOS of 20 days and average healthcare cost of 132,068 THB/visit (approximately 3890 USD/visit). The annual mortality rate is shown in Figure 2. The mortality rate, along with duration of hospitalization and healthcare expenditures for patients who died during hospitalization, stratified by age group, are presented in Table 2. It appeared that increasing age was correlated with increased mortality rates.

### 3.3. Factors Associated with Death at Discharge

The findings from the univariate and multivariate analyses pertaining to the factors associated with mortality at the time of discharge are delineated in Table 3. All factors identified through univariate analysis were regarded as having potential significance (*p*-value < 0.2), with the exception of a primary diagnosis of genitourinary disease, gastrointestinal disease, and others. Following the assessment of multicollinearity, the factors were incorporated into a multivariate analysis, wherein the age group of 81 years or over in comparison with the age group of 61–70 years (AOR 1.07), being female (AOR 1.13), and a primary diagnosis of respiratory disease (AOR 2.72), cardiovascular disease (AOR 1.68), musculoskeletal disease (AOR 0.61), systemic infection/septicemia (AOR 2.08), or malignancy (AOR 2.97) were found to be significantly associated with mortality at discharge.

## 4. Discussion

### 4.1. Prevalence Rates of Delirium

The prevalence rates of delirium among geriatric patients from this study exhibited a tendency to escalate over the past 5 years (2019–2023). Furthermore, these rates appeared to increase with age, as delineated in Table 1. Compared with the previous study in a similar setting, [7], the prevalence rate for the fiscal year 2010 was documented at 155.4/100,000 older persons, while the present study revealed a rate of 215.1/100,000 older persons. Due to the differences in methodology, it is difficult to directly compare these findings with the data of other nations regarding the age-adjusted prevalence and in-hospital rates per 100,000 population as determined by this study. Nonetheless, the observed upward trend in delirium prevalence rates over the preceding decade among the elderly population in Thailand aligns with findings from numerous international studies. For example, the coding of delirium among hospitalized patients in England and Scotland between the years 2012 and 2020 escalated from 1.4% to 12.2% and was particularly pronounced within the older age group [15]. Furthermore, research conducted in Peru indicated a similar upward trend in the number of delirious patients, which was greater in the older age group [16]. The possible explanation might be due to better recognition of delirium in comparison with the preceding decade in conjunction with the effect of the COVID-19 pandemic, as numerous studies demonstrated an escalation in the prevalence of delirium among hospitalized older patients diagnosed with COVID-19, which is likely attributable to hypoxia, fever, dehydration, acute inflammatory responses, the administration of particular medications, and a direct impact on the central nervous system resulting from SARS-CoV-2. Furthermore, the enforced isolation experienced by patients during their hospitalizations might be another precipitating factor for the occurrence of delirium in those admitted with COVID-19. Though respiratory tract infection emerged as the predominant primary diagnosis in this study, we did not examine the prevalence rates of patients hospitalized due to COVID-19 [17,18,19]. Within the various age groups, the prevalence rates for the fiscal years of 2019–2023 demonstrated a substantial increase compared with the fiscal year 2010, which reported only 56.5, 188.2, and 463.8 admissions per 100,000 older persons in the age groups of 61–70, 71–80, and over 80 years, respectively. These disparities may be elucidated by the increasing proportion of the aging population alongside a rise in the complexity of medical conditions. Therefore, the occurrence of delirium appeared to increase, as these factors were significant contributors to the manifestation of delirium. Nevertheless, the overall figures seem to fall short of anticipants; a plausible rationale for this discrepancy might be due to the high prevalence of under-recognition of delirium and misclassification bias. According to previous research, nurses are instrumental in identifying delirium, and the under-recognition rate among nursing staff within an intensive care unit of a tertiary care facility in Thailand was approximately 30% [20]. A study conducted in the general medical wards of another tertiary care hospital in Thailand reported rates of delirium identification by physicians and nurses as 57.3% and 61.8%, respectively [21]. A potential explanation for the failure to recognize delirium might be attributed to the atypical presentation of this condition in older adults, especially among those with dementia, coupled with an insufficient understanding of and inadequate attitude toward delirium, which ultimately contributes to a diminished ability for the effective detection and management of this condition. For example, a cross-sectional study revealed that more than 40% of nurses in a local hospital situated in Italy demonstrated insufficient understanding of delirium [22]. Additionally, a study conducted within a tertiary care facility in Thailand indicated that nurses exhibited misconceptions and a notable deficiency in knowledge concerning delirium [23]. Furthermore, a report among trainee physicians in Thailand also illustrated diminished awareness pertaining to delirium [24]. Notably, merely 14.5% of patients diagnosed with delirium had this condition documented in their discharge summaries. It is possible that physicians may possess awareness regarding the presence of delirium yet neglect to record the diagnosis officially in medical records [21].

### 4.2. Consequences of Delirium

Mortality rates associated with delirium at the time of discharge have escalated to nearly double from 2019 to 2023 (13.6% versus 23.5% per 100,000 older adults), with figures rising significantly in correlation to advancing age. When compared with the data from 2010 [7], the mortality rates for each age group were recorded at 2.7, 7.4, and 16.8 cases/100,000 older persons, while the results of this study indicated substantially elevated rates compared with previous findings. In addition, the average duration of hospitalization in this study exceeded that reported in 2010 (20 days versus 16 days) [7]. Focusing on age groups, the older age group manifested a reduced duration of hospitalization. This finding contrasted with previous research; these studies demonstrated that advancing age significantly correlates with prolonged hospital stays that are attributable to a multitude of factors, including but not limited to frailty, insufficient social support, and specific health conditions [25,26,27]. The possible reasons might be due to older age-group patients in this setting potentially experiencing heightened illness severity, leading to premature mortality which would result in a reduced hospital stay. In addition, the growth of palliative care and the influence of religious beliefs in Thailand, where many patients favor dying at home rather than in a hospital setting, might result in the very elderly group opting to return home at an earlier stage compared with the younger age group [28,29]. A report highlighting the preferred place of death among older individuals in Thailand also indicated that over 70% of patients expressed a desire to pass away at home. Consequently, the results of this study might diverge from those of other studies owing to variances in cultural contexts [30,31]. In terms of hospital healthcare costs within a hospital setting, the average cost per visit was consistent with data reported in 2010 (50,060.9 versus 53,174 THB) and appeared to diminish with advancing age. This phenomenon may be explicable by the reduced duration of hospitalization observed in the older age group, which consequently led to lower healthcare costs [7].

### 4.3. Factors Associated with Death at Discharge

Several studies reported that delirium increased mortality rates at the point of discharge [6,32]. This study identified predictive factors that were linked to mortality. An advancing age of 81 years or older was associated with a heightened mortality rate at discharge in contrast with individuals aged 61–70 years. Our findings aligned with a previous study that found patients who were 65 years of age or older exhibited higher mortality rates at discharge in comparison with their counterparts who were under the age of 65, with the very-old group having a greater risk than the young-old group (hazard ratio of 2.62 versus 1.09) [30]. Aging is recognized as an independent determinant of mortality within the hospital setting at discharge, as this population is vulnerable to experiencing physical and cognitive declines subsequent to critical illness, abrupt clinical deterioration accompanied by alterations in care objectives, and restriction of treatment options [33]. However, within the demographic of the elderly population, the age group of 71–80 years did not serve as an independent variable. Potential explanations may reside in the variations inherent to the sampled populations. Being female was another significant factor related to death at hospital discharge. These findings were incongruent with prior research studies. Several investigations indicated that females exhibited a heightened incidence of postoperative delirium, particularly in the context of cardiac surgeries. In contrast, males displayed a greater prevalence of delirium within the intensive care unit environment [34,35]. These observed discrepancies imply a multifaceted interaction among various determinants, encompassing both biological and sociocultural factors that were not evaluated in the current study.

Primary diagnosis of various health conditions showed significant risks, including respiratory disease, cardiovascular disease, systemic infection/septicemia, and malignancy. These findings were consistent with the prior study, indicating that specific health conditions like respiratory infection and malignancy were related to the 1-year mortality rate among hospitalized patients experiencing delirium [36]. Given the multifactorial etiology of delirium, these particular health conditions indicate physiologic disturbance, heightened susceptibility to pharmacological interventions, and increased risk of infections, all of which may complicate patients’ medical conditions and lead to heightened mortality at hospital discharge [3,4,5,6]. Musculoskeletal disease appeared as a protective factor related to mortality at hospital discharge (AOR 0.61); the possible reason for this might be attributed to including a diverse spectrum of disease severities among older hospitalized patients with this condition in this study. The management of uncomplicated musculoskeletal disease has the potential to facilitate the recovery of patients; however, frail, elderly individuals presenting with hip fractures and delirium exhibited an increased mortality risk at discharge [37].

Our findings reported the national data concerning the burdens imposed on older patients afflicted with delirium over the past 5 years and compared it with the national data from the preceding decade. It underscored the significant escalation in prevalence and mortality rates among older patients suffering from delirium throughout the last 10 years. Given that delirium is a modifiable and preventable syndrome and is likely a marker for more severe illnesses or medical complications during hospitalization [38], there exists an urgent imperative for the development of specialized delirium education programs directed toward healthcare providers, as well as the implementation of delirium prevention and early management programs, which should include the encouragement of utilizing valid and reliable diagnostic tests for delirium in every hospital level, such as the Thai version of the CAM and the 4AT test [8,9,10]. Furthermore, hospital policies should adopt delirium prevention initiatives specifically for hospitalized older patients, particularly those within high-risk groups for mortality at discharge, which include older patients admitted with respiratory diseases, genitourinary disorders, cardiovascular conditions, gastrointestinal diseases, systemic infections/septicemia, and malignancies. The timely execution of these strategies has the potential to alleviate the adverse outcomes associated with delirium.

There were several limitations associated with this study. Initially, due to its retrospective design, certain factors and outcomes were restricted in terms of analysis, including prevalence rates of delirium, severity of illnesses at presentation, presence of intensive care unit admission, medication use, predisposing factors (such as preexisting cognitive status and sensory deficits and comorbidities), functional outcomes, cognitive outcomes, caregiver burden, and post-hospitalization costs. Additionally, it limited the ability to infer causality between delirium and mortality outcomes. Secondly, the recorded prevalence rates appeared lower than anticipated, possibly due to misclassification stemming from underdiagnosis or under-coding. This could affect the accuracy of the results obtained.

## 5. Conclusions

The prevalence rates of delirium in geriatric patients during hospitalization, as well as mortality rates, exhibit an upward trend concomitant with aging. Individuals aged 81 years or older, when compared with those aged 61 to 70 years, along with the gender variable of being female and specific primary diagnoses of respiratory disease, cardiovascular disease, systemic infection/septicemia, or cancer, were found to be significantly correlated with mortality at the time of discharge.

## Figures and Tables

**Table 1:** Prevalence rates of older patients, average length of stay, and average healthcare cost by age from 2019–2023.

Year	Age Group (Years)	Prevalence Rates	LOS (Day)	Cost (THB)
2019	61–70	79.7	11	49,742
	71–80	252.5	10	39,581
	81+	560.6	9	32,020
2020	61–70	84.2	11	56,728
	71–80	260.5	10	43,148
	81+	583.1	9	33,868
2021	61–70	95.6	12	77,971
	71–80	279.3	10	59,018
	81+	607.1	9	43,854
2022	61–70	101.7	12	71,901
	71–80	301.9	11	58,701
	81+	686.4	9	47,498
2023	61–70	110.0	11	65,202
	71–80	330.7	10	49,857
	81+	767.1	9	38,640

Note: LOS; length of stay; prevalence rates were calculated per 100,000 older persons in the same age group.

**Table 2:** Mortality rates of older patients, average length of stay, and average healthcare cost by age from 2019–2023.

Year	Age Group	Mortality Rates	LOS (Day)	Cost (THB)
2019	61–70	6.7	23	131,252
	71–80	18.4	26	116,069
	81+	35.9	18	85,065
2020	61–70	6.5	21	137,646
	71–80	17.4	20	107,906
	81+	37.9	20	96,861
2021	61–70	9.8	19	181,056
	71–80	23.6	19	157,977
	81+	51.0	18	124,791
2022	61–70	10.2	22	175,667
	71–80	25.7	22	162,935
	81+	62.9	18	124,559
2023	61–70	10.9	21	152,229
	71–80	27.8	22	132,886
	81+	68.1	16	89,274

Note: LOS, length of stay; mortality rates were calculated per 100,000 older persons in the same age group.

**Table 3:** Factors associated with death at discharge.

Factors	Crude OR (95%CI)	*p*-Value	Adjusted OR 95% CI	*p*-Value
Age (year)				
61–70	-	-	1	-
71–80	0.86 (0.82–0.90)	<0.05	0.95 (0.90–1.00)	0.06
81+	0.87 (0.83–0.92)	<0.05	1.07 (1.01–1.13)	<0.05
Sex				
Men	-	-	-	-
Women	1.38 (1.33–1.44)	<0.05	1.13 (1.08–1.18)	<0.05
Primary diagnosis				
Respiratory disease	2.55 (2.45–2.66)	<0.05	2.72 (2.55–2.91)	<0.05
Neurological disease	0.62 (0.58–0.67)	<0.05	0.99 (0.91–1.09)	0.91
Genitourinary disease	0.98 (0.91–1.04)	0.47	-	-
Cardiovascular disease	1.27 (1.21–1.34)	<0.05	1.68 (1.56–1.81)	<0.05
Gastrointestinal disease	0.98 (0.91–1.05)	0.57	-	-
Musculoskeletal disease	0.58 (0.53–0.64)	<0.05	0.61 (0.55–0.68)	<0.05
Metabolic disease	0.51 (0.46–0.55)	<0.05	1.02 (0.92–1.12)	0.74
Systemic infection/septicemia	1.28 (1.17–1.43)	<0.05	2.08 (1.86–2.33)	<0.05
Malignancy	2.87 (2.69–3.07)	<0.05	2.97 (2.72–3.24)	<0.05

Note: OR, odds ratio; CI, confidence interval.

## Data Availability

Raw data were generated at the National Health Security Office (NHSO), Bangkok. Derived data supporting the findings of this study are available from the corresponding author (PL) on request.
